# Phylogenomic analysis unravels evolution of yellow fever virus within hosts

**DOI:** 10.1371/journal.pntd.0006738

**Published:** 2018-09-06

**Authors:** Chen Chen, Dong Jiang, Ming Ni, Jing Li, Zhihai Chen, Jingyuan Liu, Hanhui Ye, Gary Wong, Wei Li, Yuanyuan Zhang, Beibei Wang, Yuhai Bi, Danying Chen, Ping Zhang, Xuesen Zhao, Yaxian Kong, Weifeng Shi, Pengcheng Du, Gengfu Xiao, Juncai Ma, George F. Gao, Jie Cui, Fujie Zhang, Wenjun Liu, Xiaochen Bo, Ang Li, Hui Zeng, Di Liu

**Affiliations:** 1 Institute of Infectious Diseases, Beijing Ditan Hospital, Capital Medical University, Beijing, China; 2 Beijing Key Laboratory of Emerging Infectious Diseases, Beijing, China; 3 Beijing Institute of Radiation Medicine, Beijing, China; 4 Institute of Microbiology, Chinese Academy of Sciences, Beijing, China; 5 Clinical Center for Infectious Diseases, Beijing Ditan Hospital, Capital Medical University, Beijing, China; 6 Department of Intensive Care Unit, Beijing Ditan Hospital, Capital Medical University, Beijing, China; 7 Fuzhou Infectious Disease Hospital of Fujian Medical University, Fuzhou, China; 8 Institute of Pathogen Biology, Taishan Medical College, Taian, China; 9 Wuhan Institute of Virology, Chinese Academy of Sciences, Wuhan, China; 10 Medical School, University of Chinese Academy of Sciences, Beijing, China; 11 Chinese Center for Disease Control and Prevention (China CDC), Beijing, China; Center for Disease Control and Prevention, UNITED STATES

## Abstract

The yellow fever virus (YFV) recently reemerged in the large outbreaks in Africa and Brazil, and the first imported patients into Asia have recalled the concerns of YFV evolution. Here we show phylogenomics of YFV with serial clinical samples of the 2016 YFV infections. Phylogenetics exhibited that the 2016 strains were close to Angola 1971 strains and only three amino acid changes presented new to other lineages. Deep sequencing of viral genomes discovered 101 intrahost single nucleotide variations (iSNVs) and 234 single nucleotide polymorphisms (SNPs). Analysis of iSNV distribution and mutated allele frequency revealed that the coding regions were under purifying selection. Comparison of the evolutionary rates estimated by iSNV and SNP showed that the intrahost rate was ~2.25 times higher than the epidemic rate, and both rates were higher than the long-term YFV substitution rate, as expected. In addition, the result also hinted that short viremia duration of YFV might further hinder the evolution of YFV.

## Author summary

The first importation of infections into China in 2016 and the following outbreaks in Africa and Brazil of yellow fever virus (YFV) have raised again the concerns of the potential viral spread into new territories. In this study, we aimed to know the evolution dynamics of YFV by using intrahost phylogenomics and to assess the risk of virus epidemics. Through deep sequencing of consecutive samples from 12 patients, we identified hundreds of genomic variations (iSNVs and SNPs), and noticed the nearly linear accumulation of variations within individuals. The estimated evolutionary rate within host is much higher than the epidemic evolutionary rate. In comparison with Dengue virus (DENV) and Zika virus (ZIKV), which share similar host vectors (Aedes spp.), life cycles, mutation rates and replication strategies to YFV, the lower epidemic evolutionary rate of YFV might have been hindered by the shorter viremia duration, which decreased the accumulated variations to get into the transmission cycle.

## Introduction

Yellow fever is a notorious mosquito-borne viral disease emerged during the 15th-19th centuries in the Americas, Africa and Europe, causing severe hemorrhagic fever and liver injury with high mortality rates. Although control of mosquitoes and the use of the live-attenuated yellow fever virus (YFV)-17D vaccine strain have effectively prevented and controlled the epidemics, YFV is estimated to cause approximately 30,000 deaths out of 200,000 infections annually worldwide, mostly in Africa (https://www.cdc.gov, last accessed 10^th^ April, 2018). The etiological agent of the disease, YFV, is a single-stranded, positive-sense RNA virus with a ~11 kb genome in length. The virus is frequently transmitted between nonhuman primates and mosquitoes in African and American jungles, known as the sylvatic cycle [1]. Occasionally, YFV can escape from the sylvatic cycle to infect humans, with subsequent transmission between mosquitoes and humans, forming the urban cycle. Historically, YFV was believed to have originated from Central or East Africa, and transmitted to America during the slave trade [2, 3]. Currently, YFV in Africa and America are classified into seven genotypes, with two in South America, two in West Africa, and three in East and Central Africa [2, 4, 5].

In January 2016, the Ministry of Health of Angola notified the World Health Organization (WHO) of a yellow fever disease outbreak: as of October 28^th^, 4,347 suspected cases, including 377 deaths, were reported from all 18 provinces of Angola. Outbreaks were also reported simultaneously from the Democratic Republic of Congo and Uganda [6]. In February 2018, 464 confirmed human cases of yellow fever have also been reported in Brazil, with 154 deaths (http://www.who.int, last accessed 10^th^ April, 2018). Meanwhile, epizootics have expanded to areas previously not considered at risk for yellow fever (http://www.who.int/csr/don/27-february-2018-yellow-fever-brazil/en/, last accessed on 10^th^ April, 2018). All these recalled the concerns of the evolution of YFV. During the Angolan outbreak, YFV infections were detected in Chinese workers returning to China from Angola [7–9], marking the first time YFV infections were documented in Asia. By using the consecutive samples from those imported YFV patients, we unraveled the intrahost and epidemic evolutionary dynamics of YFV.

## Results

### Deep sequencing of YFV genomes from clinical samples

**Fig 1:**
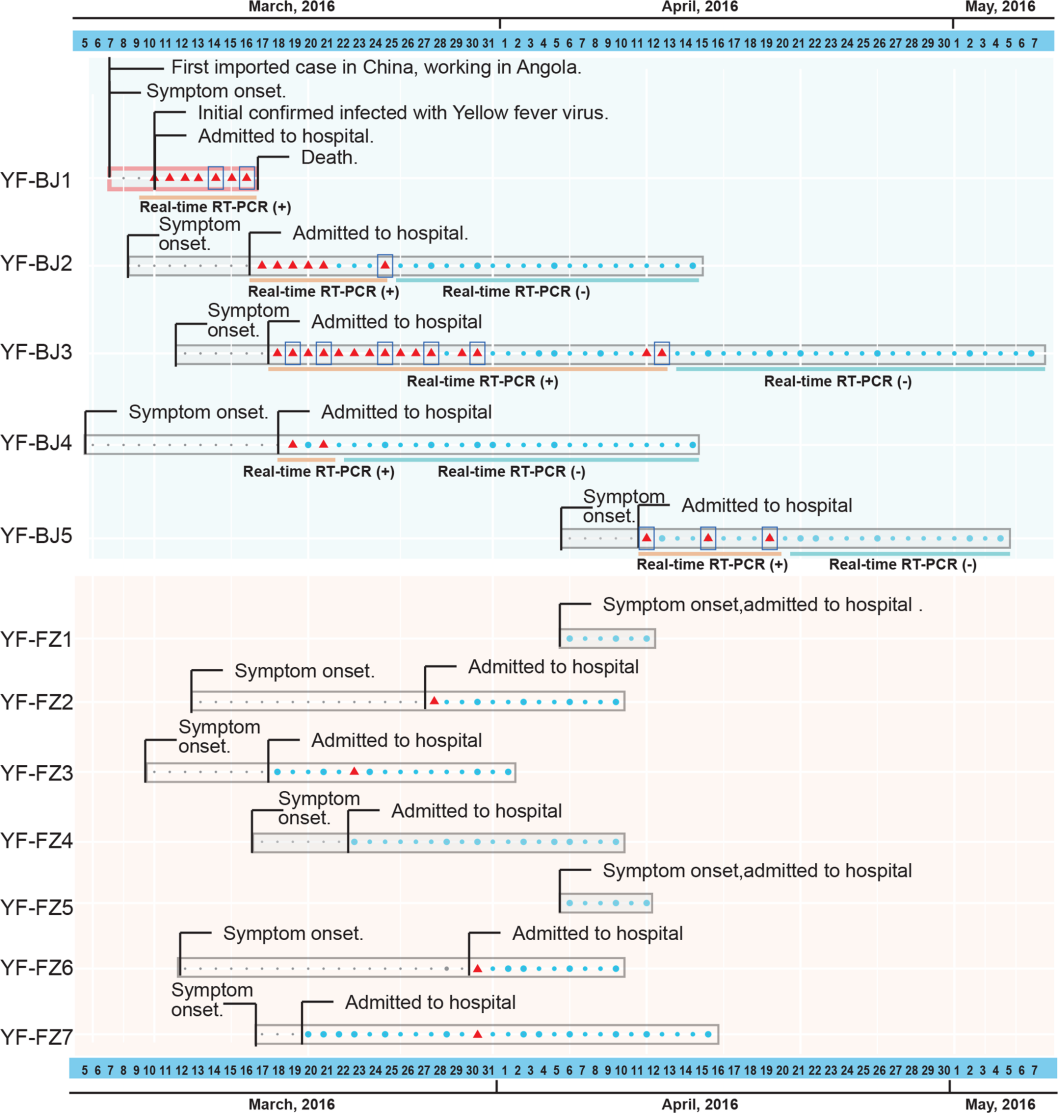
Cases of twelve documented returning YFV patients in China by date in the outbreak of Angola from March to May 2016. Timeline of events for each patient from symptom onset to the leaving from hospital are shown with different molecular detection methods and results. Dots denote that we did not get any positive molecular detection result in the time point, grey (before admitted to hospital) and blue (after admitted to hospital). The larger size dots means that we performed Real-time PCR detections in that point. The red triangles denote the sample have been detected by PCR and Real-time PCR with positive results. Blue boxes denote the samples that were subjected to NGS analysis at the time points.

We collected samples from twelve out of thirteen YFV patients in China, sequenced the viral genomes, and performed analyses on phylogenetics, sequence comparison, and intra-host dynamics. Of the 12 patients, two had severe disease, in which one survived (YF-BJ3) and one died (YF-BJ1), and the remaining patients displayed mild symptoms (*Table 1*). We tested blood samples of these 12 patients from the first day of admittance to the hospital by real-time reverse transcription PCR (RT-PCR), and four patients (YF-BJ1, YF-BJ2, YF-BJ3, and YF-BJ5) were positive. YFV RNA fragments could be detected in serum until 9, 12, 10 and 6 days, respectively, after the onset of symptoms (*Table 1, and Fig 1*). Urine samples from all patients were also tested by real-time RT-PCR and all were YFV-positive. Higher viral RNA loads were observed in the urine compared to the blood samples, except for those from the non-survivor (*Table 1*). The virus persisted in urine samples for at least 15 days after the onset of symptoms in survivors. Specifically, the urine sample from patient YF-BJ3 was still PCR-positive when tested 32 days after the onset of symptoms (*Table 1 and Fig 1*). We then sequenced all available YFV-positive samples by using total RNA sequencing, amplicon sequencing and/or Sanger sequencing (*Table 1 and* *S*1 *Table*). In total, nearly complete virus genomes (>10,222 bp) from 9 patients, and deep-sequenced genome datasets (average sequence depth 34,978x) from 3 patients covering 12 time points were obtained (*Table 1*).

**Table 1:** Patient information, viral loads and genome sequencing in urine samples.

Patient ID	Group	Outcome	Days after onset	Blood virus (Ct)*	Urine virus (Ct)*	Sequencing Method†	# Total Reads	# Mapped Reads	Sequencing Depth (x1000)	Genome Coverage (bp)
YF-BJ1	Severe	Death	7	31	35.00	AS	1,695,368	1,509,993	30.98	10,732
			8	32	33.50	AS	1,661,085	953,063	19.67	10,650
			9	30	34.42	AS	3,303,973	1,952,626	35.68	10,725
YF-BJ3	Severe	Discharged	8	27.6	25.60	AS	1,451,105	1,354,090	27.59	10,650
			10	27.9	25.32	AS	1,941,400	2,369,835	18.97	10,393
			14	-	32.85	AS	4,034,937	2,896,728	55.05	10,405
			17	-	31.66	AS	1,998,163	1,861,631	36.67	10,397
			20	-	34.00	AS	1,920,032	1,416,518	28.89	10,393
			32	-	39.83	AS	1,424,735	1,014,331	21.80	10,650
YF-BJ5	Mild	Discharged	6	23.84	23.77	AS	2,001,845	1,866,672	37.82	9,918
			10	-	35.42	AS	1,684,026	1,014,398	18.54	10,674
			14	-	34.23	AS	2,090,128	840,862	16.56	10,394
YF-BJ2	Mild	Discharged	16	-	30.95	AS	1,924,145	1,467,048	30.21	10,398
YF-BJ4	Mild	Discharged	19	-	35.86	TS	56,728,408	873	0.02	10,393
YF-FZ1§	Mild	Discharged	6	-	-	TS	77,238,978	129	0.00	1,012
YF-FZ2¶	Mild	Discharged	16	-	39.37	TS	58,570,126	195	0.00	10,243
YF-FZ3§	Mild	Discharged	14	-	39.40	TS	50,462,246	68	0.00	839
YF-FZ4¶	Mild	Discharged	7	-	-	TS	-	-	-	10,222
YF-FZ5§	Mild	Discharged	6	-	-	TS	56,605,654	68	0.00	2,795
YF-FZ6¶	Mild	Discharged	20	-	32.44	TS	51,627,058	4,722	0.11	10,838
YF-FZ7¶	Mild	Discharged	15	-	33.96	TS	64,345,650	3,945	0.09	10,311

* “-”, Negative results (Ct value >45).

† AS, amplicon sequencing; TS, total RNA sequencing.

¶ Genome sequence assembled with Sanger sequencing data.

§ Genome sequence failed to assemble.

### Phylogenetics and sequence comparison of YFV genome

**Fig 2:**
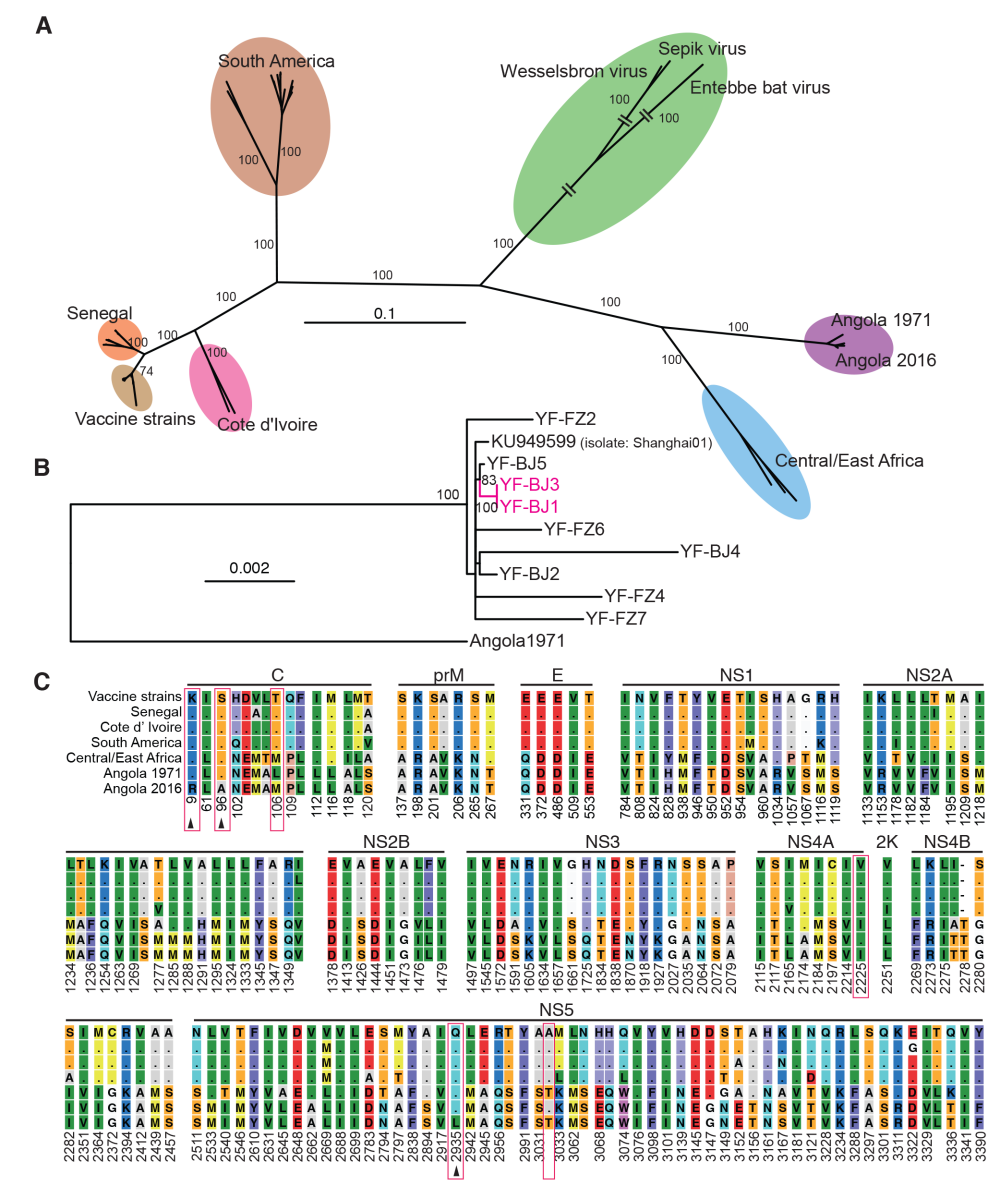
Phylogenetics and amino acids comparison of YFV viruses. (A) Maximum-likelihood phylogenetic tree of YFV genome sequences (the first 130 bps in 5’-UTR and the last 370 bps in 3’-UTR sequences are excluded). Bootstrap support values are shown along the branches. Wesselsbron virus, Sepik virus and Entebbe bat virus were used as outgroup. (B) Maximum-likelihood phylogenic tree of YFV genomes of Angola 2016 strains. Viruses from patients with severe disease are highlighted in magenta. Angola 1971(GenBank accession AY968064) was used as outgroup. Bootstrap support values (≥70) are shown. (C) Comparison of amino acids of YFV consensus sequence of each lineage in (A). Identical amino acid to vaccine strain (17D) lineage is denoted as a dot. The amino acid sites that were different in Angola 2016 and 1971, are highlight with red rectangles. Novel amino acid substitutions of Angola 2016 are denoted by arrowheads.

We performed phylogenetic analysis of the coding region of YFVs (S2 *Table*), and found that YFV sequences from the returning workers in 2016 outbreak closely clustered with the 1971 Angola strains (*Fig 2A*). Of note, the vaccine strain YF-17D and its derivatives are located far from the Angola strains on the phylogenetic tree. A closer inspection of the Angola strains shows that the viruses in the 2016 outbreak are likely from a single origin and genome sequences from both severely ill patients are closely clustered (*Fig 2B*). There are 188 nucleotide substitutions between the 1971 and 2016 consensus sequences and 6 of them are nonsynonymous substitutions. Comparison of the YFV polypeptides shows that only three amino acid changes that appear to be specific to the Angola 2016 strains (*Fig 2C*). Two are in the capsid protein and one in NS5, the RNA-dependent RNA polymerase. The above data are consistent with previous studies using partial or whole genomes showing that YFV exhibits a slow evolutionary rate [2, 4, 10].

### Intrahost genomic variations

**Fig 3:**
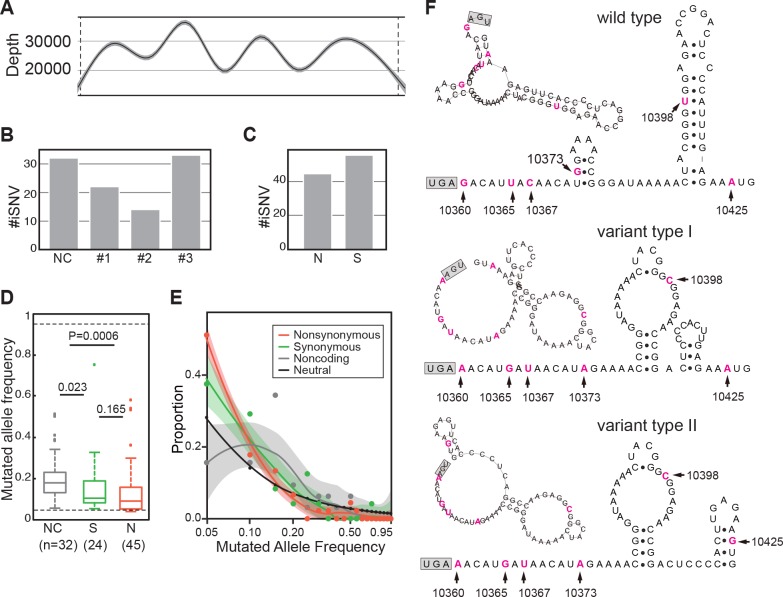
The iSNVs of Angola 2016 YFVs from clinical samples. (A) Sequencing depth across the sequenced genomes. The x-axis represents the YFV genome, with the ORF boundaries indicated by vertical dashed lines. The sequencing depth smoothed by locally weighted smooth regression (LOESS) is shown by the black curve, with 95% confidence interval as shown by shadow. (B) Numbers of iSNVs at non-coding (NC) regions and codon positions of the ORF. (C) Numbers of nonsynonymous (N) and synonymous (S) iSNVs of the ORF. (D) Box plots of the mutated allele frequencies (MuAFs) for non-coding, synonymous and nonsynonymous iSNVs. The MuAF values of three kinds of iSNVs are compared to each other with the Wilcoxon rank-sum test. Dashed lines denote the boundaries of MuAFs in iSNV identification. Boxes denote the interquartile range (IQR) between the first and third quartiles. Lines inside the boxes indicate the median, and the lines outside boxes represent values within 1.5 times the IRQ. Outliers are denoted as dots. (E) Comparison of MuAF spectra of non-coding, synonymous and nonsynonymous iSNVs. MuAFs (dots) are shown with LOESS lines (95% confidence interval in shadow). Expectation under neutral selection is shown by a black line. (F) Prediction of RNA secondary structures of 3’ UTR (with 68 bp downstream of the stop codon). The earliest type of Angola 2016 (wildtype) and two nucleotides variant types are illustrated. Six variation sites are highlighted in magenta. The stop codon is in the gray box.

For the deep-sequenced samples, we identified the iSNVs by using methods in a previous study for Ebola virus [11]. The mean sequencing depth of genomic regions is between 16,558x and 37,822x (*Fig 3A*), and a total of 101 iSNV sites were discovered, including 69 in the coding region and 32 in non-coding regions. In each iSNV sites, we only found two types of nucleotides. Taken that sequencing errors are generated randomly and may result in multi-nucleotide heterogeneity in a single site, it is unlikely those iSNVs were the results of sequencing bias. Fewer iSNVs appeared in the 1^st^ and 2^nd^ codon positions than the 3^rd^ codon position (*Fig 3B*) and fewer non-synonymous than synonymous iSNVs (*Fig 3C*), implying that the coding region is generally under purifying selection. We plotted the distributions of all mutated allele frequencies of the iSNVs (*Fig 3D and 3E*). The mean mutated allele frequency of non-coding iSNVs was at 0.18, while that of synonymous and non-synonymous iSNVs was 0.12 and 0.09, respectively (*Fig 3D*). Among them, non-synonymous iSNV is significantly lower than that in non-coding regions (P = 0.02). The distribution of non-coding iSNVs is close to the expected neutrality, whereas curves of synonymous and nonsynonymous iSNVs have higher portions of iSNVs in the area of low mutated allele frequencies (*Fig 3E*). This further supports the notion that the coding region was under purifying selection. Additionally, we discovered two variant types at the 3’ untranslated region (UTR), one with 5 concurrent iSNVs (G10360A, U10365G, C10367U, G10373A, and U10398C) and the other having an additional iSNV (A10425G) (*Fig 3F*). Phasing analysis reveals that these iSNVs tend to be concurrent in the same reads (S3 *Table* *and S1 Fig*). Predicted RNA structure shows that these substitutions are likely to affect the structure of the 3’ UTR (*Fig 3F and S2 Fig*), and probably influence viral replication in hosts [12–14].

### Intrahost evolutionary dynamics

**Fig 4:**
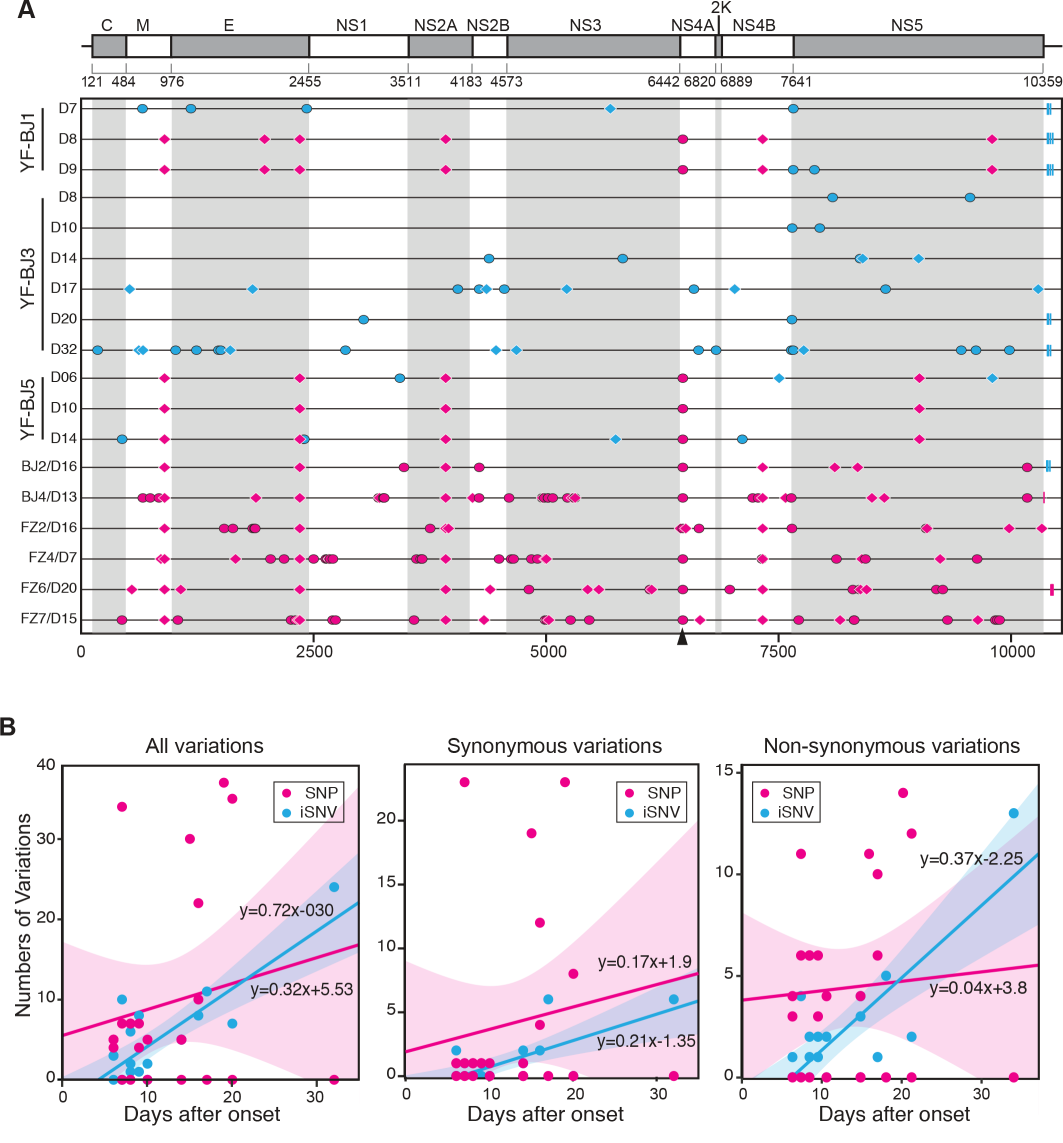
Genomic variations of Angola 2016 YFV and evolutionary model. (A) SNPs (in magenta) and iSNVs (in cyan) detected in Angola 2016 strains along the YFV genome. Non-synonymous variations are denoted by ovals, synonymous variations by diamonds, and variations in UTR by rectangles. Non-synonymous site at 6463 is denoted by arrowhead. YFV genome of patient YF-BJ1 at 7 days after the onset (YF-BJ1/7D) was used as the reference sequence (B) Linear regression of number of variations at all variations, synonymous and non-synonymous sites accumulated by days after onset. The smooth shadows denote the LOESS fit with 95% confidence interval.

Subsequently, we placed all of the polymorphic sites (SNPs and iSNVs) along viral genome (*Fig 4A*). SNPs and iSNVs described the viral difference in two levels of between and within the host, respectively. Within 65 days (from the onset of the first patient to the sample of the last patient in this study) we observed a total of 234 SNPs in 148 sites in 18 samples, including 102 synonymous and 117 nonsynonymous SNPs. A total of 138 SNPs that appear only once are scattered along the viral genome. In particular, the SNPs of 5 sites (T900A, A2352C, C3918A, G6463A, and A7320G) could be used to characterize the 2016 YFVs. All five SNPs were detected in seven patients, four SNPs (without A7320G) were detected in one patient (YF-BJ5), and only one patient (YF-BJ3) does not possess any of the above SNPs (*Fig 4A*). Of note, YF-BJ1 is likely a co-infection with more than one YFV variant, as the virus genome on day 7 does not contain any of the five SNPs, whereas all five SNPs became dominant on day 8 (*Fig 4A*). Of the five SNPs, only G6463A is nonsynonymous, resulting in an amino acid substitution from Val to Ile in NS4A.

We then compared the accumulation of polymorphic sites within patients over time (*Fig 4B*). Each sample was set to days after onset of the symptoms of the patients it belongs to. Linear regressions revealed that iSNVs and SNPs accumulated from the day of onset of symptoms at a rate of 0.72 and 0.32 variations/day, respectively. The accumulation rate of iSNV may reflect the intrahost evolution. Intriguingly, the YFV evolutionary rate within humans is roughly 2.48 x10^-2^ variations/site/year (0.72 variations/day = 0.72 x 365 / 10591 variations/site/year = 2.48 x10^-2^ variations/site/year) in our study. Of those synonymous variations, the intrahost evolutionary rate estimated by iSNV was 0.7 x10^-2^ variations/site/year; whereas the rate of nonsynonymous variations was 1.2 x10^-2^ variations/site/year. Compared to the rates estimated by SNP within these samples, the intrahost evolutionary rate of non-synonymous sites (by iSNV) is higher, while the rate of synonymous sites is slightly higher than the SNP rate.

## Discussion

The evolution of YFV is a basic question to unfold to predict the risk of the pathogens. Phylogenetic analysis suggests that the 2016 outbreak is likely the spillover of YFV viruses in Angola. The high identity between the 2016 strain and the vaccine strain YF-17D would explain the effectiveness of the current vaccines [15]. Although there is a diversity of yellow fever virus genotypes, all genotypes are indistinguishable in serological assays. There is not any evidence that the Angola outbreak was due to immune escape and it is accepted among public health professionals that low vaccine coverage in the area prior to the outbreak was the driving factor and the resulting aggressive mass vaccination campaign was vital for containing and eliminating the outbreak. However, it should be noted that the Angola lineage is clearly diversified from the 17D-lineage (*Fig 2A*). Moreover, we did notice 6 patients who had been vaccinated before [16], but the limited clinical and epidemiological data cannot tell the immune escape. Future immune escape from current YFV vaccines cannot be fully ruled out, although YFVs evolve very slowly [17]. A novel vaccine based on a Central/East African YFV isolate would be necessary as a complement vaccine to prepare against future outbreaks. Owing to the slow evolutionary rate and the past experience of using YF-17D, the novel vaccine is likely to work for another decades.

Virus evolution experienced mutation, selection within and between hosts, genetic drift, and transmission, and finally formed the genetic variants (lineages) over long-term evolutionary timescales [18]. Deep sequencing of viral genomes can provide novel insights into viral evolutionary dynamics [19, 20]. In this intra-host phylogenomic analysis, we depicted the evolutionary process of YFV from intra-host to epidemic substitutions, which were reflected by iSNV to SNP, respectively. Other than using the metric for long-term evolutionary rate, we applied the substitution/site/day to measure the intrahost evolutionary rate, which appeared more appropriate in outbreak or epidemic scenarios. Short-term rates may provide better insight into the intrahost YFV evolutionary trends (e.g., iSNV dynamics over time) relative to clinical symptomology, severity, and/or co-morbidities. However, further in vitro or in vivo studies would be needed. The estimated evolutionary rates clearly showed that the intrahost evolutionary rate is ~2.25 times higher than that of the epidemic evolutionary rate, and both are much higher (59 times and 26 times) than the substitution rate of YFV along longer evolutionary timescale [10]. The higher rate estimated by iSNV reflected purifying selection occurred, when intrahost variations were spreading out. The rates estimated by synonymous iSNV and SNP, respectively, were similar, implying that the synonymous variations were under similar evolutionary constraints during transmission. Meanwhile, only a few of the nonsynonymous variations were spread out in different individuals, and only G6463A (Val to Ile in NS4A) were detected in multiple samples.

Compard to Dengue virus (DENV) and Zika virus (ZIKV), YFV shares similar host vectors (*Aedes spp*.) and life cycles [21, 22], and has similar mutation rates and replication strategies [10, 23, 24]. However, the long-term substitution rates of DENV (0.77–0.99 x10^-3^ substitutions/site/year) and ZIKA (0.98–1.06 x10^-3^ substitutions/site/year) are higher than that of YFV [23, 25, 26]. Previous studies have posed that additional constrained selection forces are probably existed [10, 27]. Based on our results, we noticed the nearly linear accumulation of variations within individuals. Since the transmission of YFV variants needs mosquitoes as vector, the window (viremia duration) that mosquitoes could use is essential, as only the variations within this period could be brought into the transmission cycle. A wider window size would possibly allow more non-synonymous variations into the transmission cycle (Cristina Domingo, EID, 2018). Given that YFV, DENV and ZIKV have ~3, 3–7, and 4–7 days of viremia, respectively, after symptom onset (*S4 Table*), it hinted that the shorter viremia duration in YFV infections might result in the lower evolutionary rate than that of DENV or ZIKV. Most Flaviviruses are vector-borne viruses and the evolutionary rates in mammals and arthropods are different. The intrahost analysis of virus could show the short-term evolution dynamics, especially from serial samples, and this would be a helpful complement to the long-term evolution of Flaviviruses.

In addition, deep sequencing and viral genomics have provided new insights into viral evolutionary dynamics, especially in a continuous mode. Our findings strongly suggest that the urine samples should be considered for the clinical diagnosis, genomic surveillance, and large-scale screening of YFV infections. Further investigations are also encouraged to characterize the factors that maintain viremia in vector-borne diseases.

## Methods

### Ethics statement

The study was conducted in accordance with the Declaration of Helsinki and approved by the ethics committee of Beijing Ditan Hospital (DSRB 2008/00293 and DSRB 2013/00209). Clinical samples and information were obtained after written informed consents from all participants. All human subjects were adult, and if not, whether a parent or guardian of any child participant provided informed consent on their behalf.

### Patients and data collection

Twelve YFV patients returning from Angola were recruited (*Table 1*). We sampled urine and blood sample of these patients from routine examination and collected their clinical data.

### YFV diagnosis and virus detection

YFV infection was diagnosed according to the WHO guidelines (http://www.who.int/csr/disease/yellowfev/case-definition/en/, last accessed 10^th^ April, 2018), including clinical symptoms (fever, headache, jaundice, etc.) and laboratory detection of virus by using real-time RT PCR. The serum and urine samples were collected and stored at -80 ^o^C for further analyses, including real-time RT-PCR, PCR sequencing and RNA sequencing.

### Next generation sequencing

RNA from both serum and urine samples were deployed to two approaches of deep sequencing. Approach I: Total RNA sequencing. RNA was purified by Trizol (Thermo Scientific) for each sample, and then total RNA libraries were constructed with 500bps insertion size using the NEBNext UltraTM RNA Library Prep Kit (NEB, MA, USA), and then sequenced by Illumina Hi-seq platform, and generated 2 × 125 bp paired-end reads. Approach II: RNA amplicon sequencing. Two sets of YFV-specific primer pairs were designed (Set A: 5 pairs, amplicon length = 2,248 ± 168 nt; Set B: 12 pairs, amplicon length = 1,018 ± 48 nt; S5 *Table*). PCR products were sequenced by Illumina platform with 2 × 250 bp paired-end reads. Quality control and error correction were implemented according to previous report [28, 29]. The probably low quality regions in sequencing, including 1) Q<30, and 2) the first 10 bps after primers were removed from the high quality sequences, according to a previous study [28]. To minimize sequencing error that affects the accuracy of iSNV calling, we only kept the read-pair that has >100 high quality bases in both ends as clean reads. All reads were deposited in the NCBI SRA database under the accession no. SRP096859.

### Sequence alignment and phylogenetic analysis

Consensus sequences were aligned by MUSCLE [30]. Phylogenetic analyses were performed by using RAxML v8.1.6, with the GTR model of nucleotide substitution and γ-distributed rates among sites. Phylogenetic tree was constructed by maximum-likelihood using YFV genome sequences [31]. A NJ tree based on YFV genome sequences was also constructed to show the results were robust (S3 *Fig*). A total of 1,000 bootstrap replicates were performed. All SNPs were listed in S6 *Table*. The genome sequences of YF-BJ1, YF-BJ2, YF-BJ3, YF-BJ4, YF-BJ5, YF-FZ2, YF-FZ4, YF-FZ6, and YF-FZ7 were deposited in the NCBI GenBank database under the accession no. MH633684-MH633692.

### Calling of iSNVs

To minimize the potential sequencing errors generated by system, we performed the following two steps before we called iSNV, including 1) mapping the clean reads to the reference genome, 2) qualification of the samples. For mapping the clean reads to genome, we use the traditional protocol used widely. First, we mapped the clean reads with pair-ended aligned to the assembled genome from YF-BJ1/7D by using Bowtie2 v2.2.5 [32] with default parameters. SAMtools v1.2 [33] was used to generate ‘mpileup’ files with no limit for the maximum site depth. To find the potential mutation in each site, we do not use the following pipeline in SAMtools. Instead, we developed homemade PERL scripts (available at http://github.com/generality/iSNV-calling/), which were used for iSNV calling to identify all potential mutations by using the mpileup files as input. The depth of bases in each sites were used to measure the mutation rate of iSNV. Then, we selected the samples with more than 3,000 sites with a sequencing depth ≥300× as candidate samples for iSNV calling. Using this protocol, we called iSNV in 15 samples in this study (S1 *Table*), and obtained the frequencies of each allele in each genome site. Interestingly, we only found the heterogeneous nucleotides with two types in each sites. Thus, we defined the nucleotide with higher frequencies as the major allele, and fewer one as the minor allele in the following iSNV calling.

The iSNV calling was according to the site depth and strand bias with following: 1) remove the ambiguous iSNV and keep the following iSNVs: (1.1) minor allele frequency of ≥5%, a conservative cutoff based on an error rate estimation described before [11, 28]; (1.2) depth of the minor allele of ≥15; and (1.3) strand bias of the minor allele less than tenfold. 2) In our analysis, these two types of nucleotides in each site have contained the nucleotide in the reference genome. Mutated allele frequencies were calculated by the rate of the reads numbers for mutated alleles to the references and total reads. Minor allele and Major allele frequencies were calculated by the majority nucleotides and minor nucleotides in each site respectively. 3) The effect of iSNV to the gene was calculated by the mutation whether caused the amino acid changes.

### Phasing analysis of iSNVs

The iSNVs that co-occurred in same viral haplotype(s) within quasispecies was defined as phased iSNVs. 1)We screened the iSNV sites along genome with a window of 250 bp, and then identified all the windows which probably containing a phasing iSNVs. 2) All the sequencing reads aligned to YFV genome region harboring these iSNVs were extracted. 3) The stretches of nearest iSNV were determined by the mutations in the reads aligned to the genome region with the same phasing (supporting reads > = 2). 4) The fractions of reads supporting phased and non-phased neighbor iSNVs were respectively calculated.

### Prediction of RNA secondary structures

RNA secondary structures were predicted by using RNAfold program of ViennaRNA Package 2 with default settings [34]. Nucleotide sequences started at the beginning of 3' UTRs of Angola 2016 strain. The length of window for secondary structure prediction was equal to 70 nucleotides. We also extended the additional 200 nucleotides at the end of the sequence, with a step length equal to 50.

### Accession numbers

All reads from next generation sequencing were deposited in the NCBI SRA database under the accession no. SRP096859. The genome sequences of YF-BJ1, YF-BJ2, YF-BJ3, YF-BJ4, YF-BJ5, YF-FZ2, YF-FZ4, YF-FZ6, and YF-FZ7 were deposited in the NCBI GenBank database under the accession no. MH633684-MH633692.

## Supporting information

S1 Fig Phasing analysis of iSNVs at the 3’ untranslated region (UTR). For a given sample, the sequencing reads aligned to 3’ UTR of the YFV genome were extracted to obtain the 5-mutations (at positions 10360, 10365, 10367, 10373 and 10398) or 6-mutations (10360, 10365, 10367, 10373, 10398 and 10425). The pie charts denote fractions of reads supporting wildtype 5/6-mers and phased mutant 5/6-mers (A10360G, G10365T, T10367C, A10373G, C10398T and G10425A), with read counts (or depth, x) shown in brackets. Sample IDs are indicated at the top of pie charts.
(PDF)

S2 Fig RNA secondary structure prediction of the 3’ UTR. RNA secondary structures were predicted based on sequences of YFV 3’ UTR (started from the stop codon) with gradually increased lengths. The sequences were extracted from genomes of wild type Angola 2016 strain and two variant types. Blue arrows indicate variations corresponding to the phased iSNV sites at 3’ UTR, A10360G, G10365T, T10367C, A10373G, C10398T and G10425A. The local structural alterations due to the variations are highlighted in blue shadow.
(PDF)

S3 Fig Phylogenetic tree constructed by neighbor-joining method using YFV genome sequences. Viruses from patients with severe disease are highlighted in magenta. Angola 1971(GenBank accession AY968064) was used as outgroup. Bootstrap support values over 70 are shown.
(PDF)

S1 Table Sequencing data from continuous clinical samples of YFV. (PDF)

S2 Table Public sequences used for phylogenic analysis. (PDF)

S3 Table Phasing analysis for continuous neighbor iSNV. (PDF)

S4 Table Duration of viremia and evolutionary rates of YFV, ZIKV and DENV. (PDF)

S5 Table Two primer sets used for amplicon-sequencing. (PDF)

S6 Table SNPs observed in YFV. (PDF)
