# Supplementary material for: Phylogenomic analysis unravels evolution of yellow fever virus within hosts
Source: PLoS Negl Trop Dis. 2018 Sep 6;12(9):e0006738. doi: 10.1371/journal.pntd.0006738 (PMC6143276; doi:10.1371/journal.pntd.0006738)
Supplement: S1 Fig — For a given sample, the sequencing reads aligned to 3’ UTR of the YFV genome were extracted to obtain the 5-mutations (at positions 10360, 10365, 10367, 10373 and 10398) or 6-mutations (10360, 10365, 10367, 10373, 10398 and 10425). The pie charts denote fractions of reads supporting wildtype 5/6-mers and phased mutant 5/6-mers (A10360G, G10365T, T10367C, A10373G, C10398T and G10425A), with read counts (or depth, x) shown in brackets. Sample IDs are indicated at the top of pie charts. (PDF) [file pntd.0006738.s001.pdf]

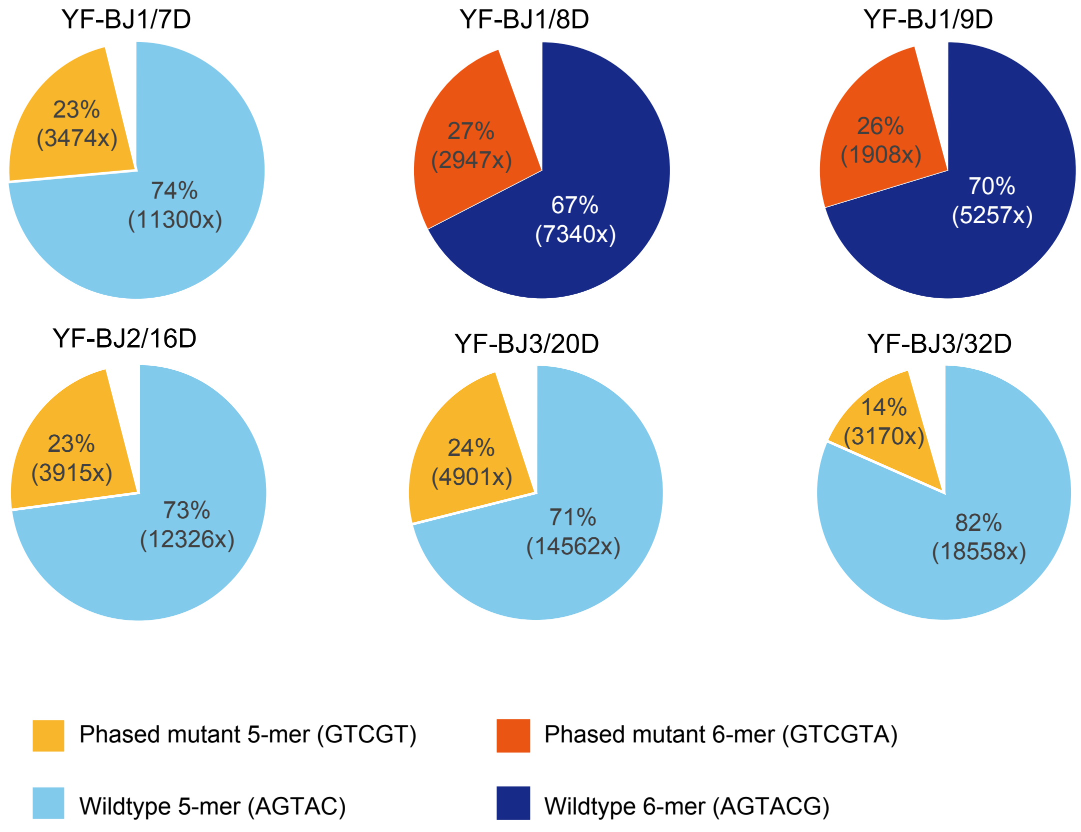

Fig S1: Phasing analysis of iSNVs at the 3' untranslated region (UTR). For a given sample, the sequencing reads aligned to 3' UTR of the YFV genome were extracted to obtain the 5-mutations (at positions 10360, 10365, 10367, 10373 and 10398) or 6-mutations (10360, 10365, 10367, 10373, 10398 and 10425). The pie charts denote fractions of reads supporting wildtype 5/6-mers and phased mutant 5/6-mers (A10360G, G10365T, T10367C, A10373G, C10398T and G10425A), with read counts (or depth, x) shown in brackets. Sample IDs are indicated at the top of pie charts.
