# Supplementary material for: Phylogenomic analysis unravels evolution of yellow fever virus within hosts
Source: PLoS Negl Trop Dis. 2018 Sep 6;12(9):e0006738. doi: 10.1371/journal.pntd.0006738 (PMC6143276; doi:10.1371/journal.pntd.0006738)
Supplement: S2 Fig — RNA secondary structures were predicted based on sequences of YFV 3’ UTR (started from the stop codon) with gradually increased lengths. The sequences were extracted from genomes of wild type Angola 2016 strain and two variant types. Blue arrows indicate variations corresponding to the phased iSNV sites at 3’ UTR, A10360G, G10365T, T10367C, A10373G, C10398T and G10425A. The local structural alterations due to the variations are highlighted in blue shadow. (PDF) [file pntd.0006738.s002.pdf]

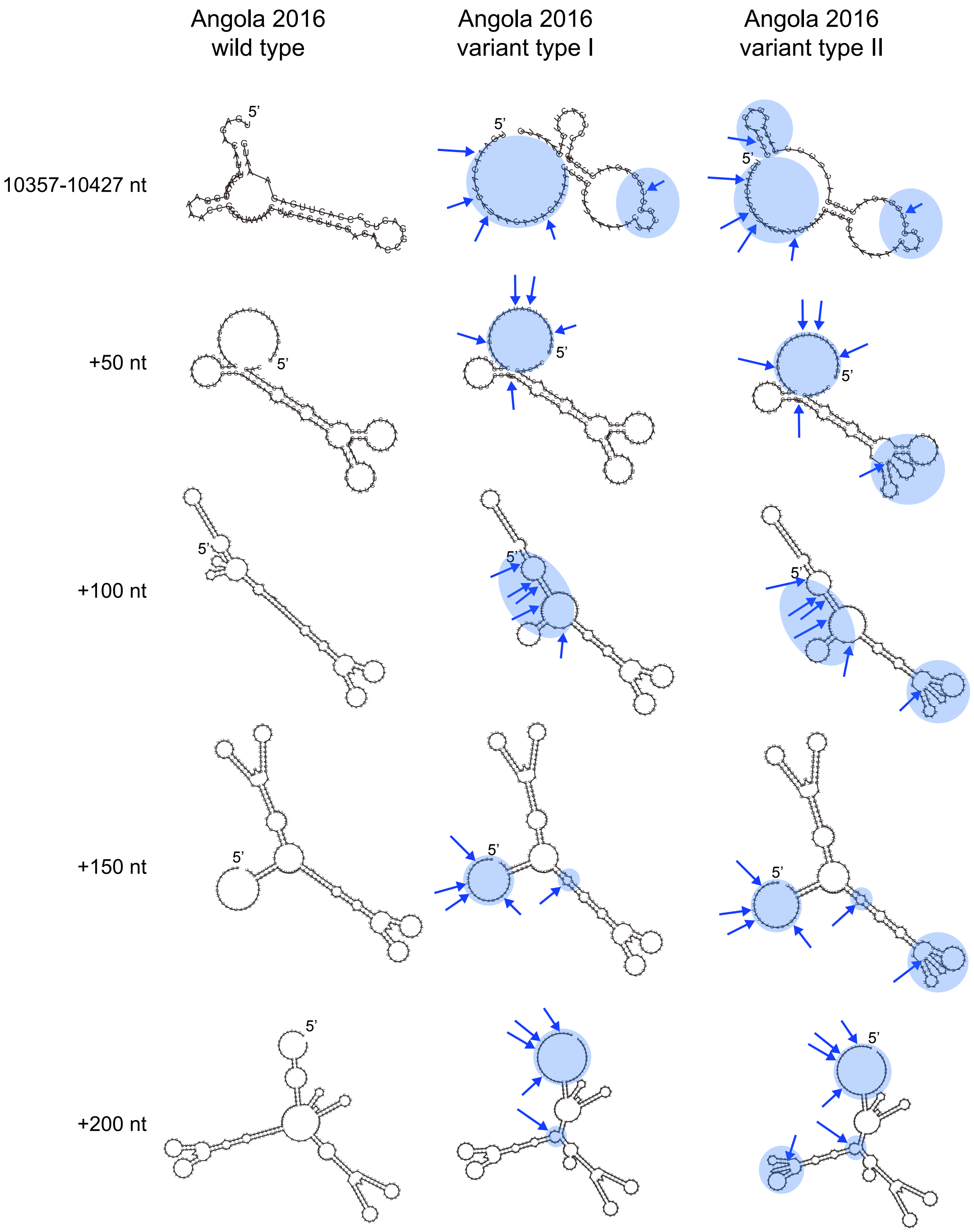

S2 Fig: RNA secondary structure prediction of the 3’ UTR. RNA secondary structures were predicted based on sequences of YFV 3’ UTR (started from the stop codon) with gradually increased lengths. The sequences were extracted from genomes of wild type Angola 2016 strain and two variant types. Blue arrows indicate variations corresponding to the phased iSNV sites at 3’ UTR, A10360G, G10365T, T10367C, A10373G, C10398T and G10425A. The local structural alterations due to the variations are highlighted in blue shadow.
