# Supplementary material for: Phylogenomic analysis unravels evolution of yellow fever virus within hosts
Source: PLoS Negl Trop Dis. 2018 Sep 6;12(9):e0006738. doi: 10.1371/journal.pntd.0006738 (PMC6143276; doi:10.1371/journal.pntd.0006738)
Supplement: S3 Fig — Viruses from patients with severe disease are highlighted in magenta. Angola 1971(GenBank accession AY968064) was used as outgroup. Bootstrap support values over 70 are shown. (PDF) [file pntd.0006738.s003.pdf]

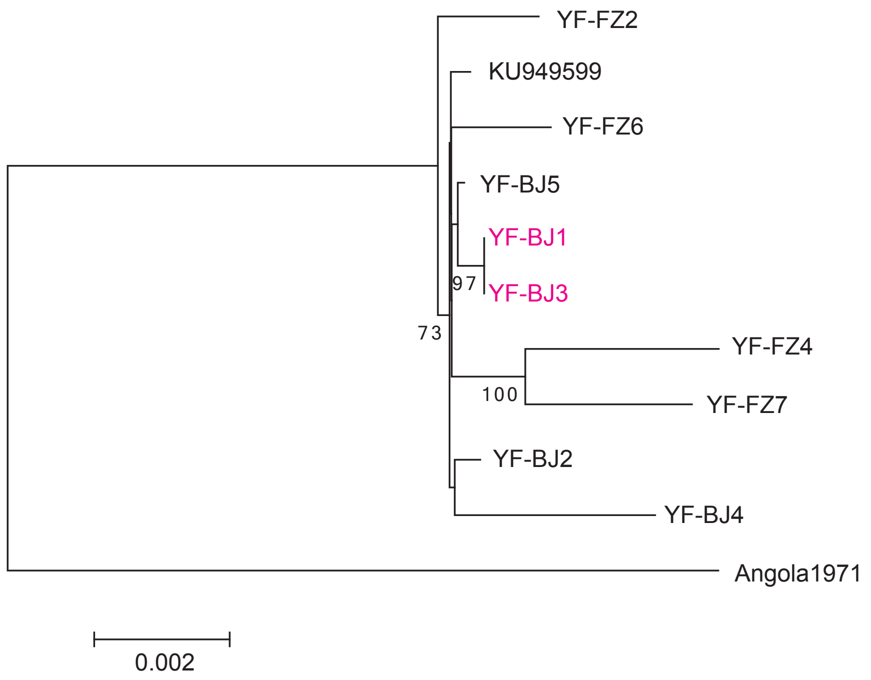

Fig S3: Phylogenetic tree constructed by neighbor-joining method using YFV genome sequences. Viruses from patients with severe disease are highlighted in magenta. Angola 1971(GenBank accession AY968064) was used as outgroup. Bootstrap support values over 70 are shown.
