# Supplementary material for: Phylogenomic analysis unravels evolution of yellow fever virus within hosts
Source: PLoS Negl Trop Dis. 2018 Sep 6;12(9):e0006738. doi: 10.1371/journal.pntd.0006738 (PMC6143276; doi:10.1371/journal.pntd.0006738)
Supplement: S1 Table — (PDF) [file pntd.0006738.s004.pdf]

Table S1. Sequencing data from continuous clinical samples of YFV

ID	Sample Name	Amplicon sequencing				Total RNA sequencing			Total Genome coverage assembled with NGS and Sanger data
		Reads #	Coverage	Mean Depth of Coverage	Primer Set(Failed paires)	Reads #	Reads # matched to YFV	Matched percentage	
1	YF-BJ1/7D	1,940,500	10,805	30,981.16	Set2	-	-	-	10,732
2	YF-BJ1/8D	1,661,085	10,649	19,665.84	Set2	-	-	-	10,650
3	YF-BJ1/9D	3,303,973	10,724	35,682.06	S076, set 2； S052,set 1	-	-	-	10,725
4	YF-BJ1/6Db*	1,665,809	5,828	42,634.84	Set2(Failed, 2,3,6,9,11,12)	-	-	-	5,830
5	YF-BJ1/7Db*	1,739,605	10,004	26,201.57	Set2(Failed,12)	-	-	-	10,005
6	YF-BJ1/8Db*	1,870,603	10,004	29,883.67	Set2(Failed,12)	-	-	-	10,005
7	YF-BJ1/9Db*	1,548,543	5,915	43,807.15	Set2(Failed,3,5,6,8,9,12)	-	-	-	5,916
8	YF-BJ2/16D	1,924,145	10,649	30,205.37	Set2	75,716,292	380	0.001%	10,650
9	YF-BJ3/8D	1,451,105	10,392	27,585.82	Set1	6,577,410	5,052	0.086%	10,393
10	YF-BJ3/10D	2,736,970	10,472	65,340.19	Set2	2,756,328	28,183	1.246%	10,405
11	YF-BJ3/14D	4,034,937	10,396	55,045.55	Set1	3,001,579	5,294	0.202%	10,397
12	YF-BJ3/17D	1,998,163	10,392	36,668.93	Set1	6,602,261	2,184	0.039%	10,393
13	YF-BJ3/20D	1,920,032	10,649	28,888.97	Set2	-	-	-	10,650
14	YF-BJ3/32D	1,424,735	9,917	21,802.37	Set2	-	-	-	9,918
15	YF-BJ4/19D	-	-	-	-	56,728,408	873	0.002%	10,674
16	YF-BJ5/6D	2,001,845	10,392	37,822.96	Set1	-	-	-	10,394
17	YF-BJ5/10D	1,684,026	10,395	18,544.42	Set1	-	-	-	10,398
18	YF-BJ5/14D	2,090,128	10,392	16,558.65	Set1	-	-	-	10,393
19	YF-FZ1/6D	-	-	-	-	77,238,978	129	0.000%	1,012
20	YF-FZ2/16D	-	-	-	-	58,570,126	195	0.000%	10,243
21	YF-FZ3/14D	-	-	-	-	50,462,246	68	0.000%	839
22	YF-FZ4/7D	-	-	-	-	-	-	-	10,222
23	YF-FZ5/6D	-	-	-	-	56,605,654	68	0.000%	2,795
24	YF-FZ6/20D	-	-	-	-	51,627,058	4,722	0.010%	10,838
25	YF-FZ7/15D	-	-	-	-	64,345,650	3,945	0.007%	10,311

*Sample from blood
