# Supplementary material for: Phylogenomic analysis unravels evolution of yellow fever virus within hosts
Source: PLoS Negl Trop Dis. 2018 Sep 6;12(9):e0006738. doi: 10.1371/journal.pntd.0006738 (PMC6143276; doi:10.1371/journal.pntd.0006738)
Supplement: S2 Table — (PDF) [file pntd.0006738.s005.pdf]

Table S2. Public sequences used for phylogenic analysis

StrainName	Source of isolation	Place of isolation	Date of isolation	Accession number
Angola 71	Human	Angola	1971	AY968064
Couma-Ethiopia 61	Human	Ethiopia	1961	DQ235229
Uganda 48	Human	Uganda	1948	AY968065
Uganda2010	Human	Uganda	2010	JN620362
Ivory_Coast99	Human	Ivory Coast	1999	AY603338
HD 117294	Human	Koungheul/Senegal	1995	JX898868
Gambia 01	Human	Gambia	2001	AY572535
ArD 149214	*Aedes furcifer*	Koungheul/Senegal	2000	JX898873
ArD 149194	Aedes taylori	Koungheul/Senegal	2000	JX898874
ArD 121040	*Aedes furcifer*	Koungheul/Senegal	1996	JX898870
ArD 149815	*Aedes furcifer*	Koungheul/Senegal	2000	JX898875
AR378600	*Haemagogus sp.*	Uruaçu, GO/Brazilian	1980	JF912179
ArD 181564	*Aedes luteocephalus*	Koungheul/Senegal	2005	JX898880
ArD 156468	*Aedes furcifer*	Koungheul/Senegal	2001	JX898876
ArD 181464	*Aedes furcifer*	Koungheul/Senegal	2005	JX898877
ArD 181250	*Aedes furcifer*	Koungheul/Senegal	2005	JX898878
ArD 181676	*Aedes taylori*	Koungheul/Senegal	2005	JX898879
ArD 181439	*Aedes luteocephalus*	Koungheul/Senegal	2005	JX898881
BeH463676	Human	Breves, PA/Brazilian	1987	JF912184
BeAR513008	Human	Sidrolândia, MS/Brazilian	1992	JF912185
BeH622205	Human	Uruaçu, GO/Brazilian	2000	JF912187
BeH622493	Human	Alto Paraíso, GO/Brazilian	2000	JF912188/Asibi
Asibi	Human	Ghana	1927	AY640589
Asibi	Vaccine	Ghana	1972	KF769016
DakArAmt7	*Aedes africanus*	Cote d'Ivoire	1973	JX898869
French viscerotropic	Vaccine	-	-	U21056
BeH394880	Human	C. do Araguaia, PA	1981	JF912180
ArD 114972	*Aedes aegyptia*	Koungheul/Senegal	1995	JX898872
ArD 114896	*Aedes aegyptia*	Koungheul/Senegal	1995	JX898871
French neurotropic	Vaccine	-	-	U21055
Trinidad1979	Human	Trinidad	1979	AF094612
BeH423602	Human	São Domingos do Capim, PA	1984	JF912183
BeAR646536	*H. Leucocelaenus*	S.A. Missões, RS	2001	JF912189
BeH526722	Human	Arinos, MG	1994	JF912186
BeH655417	Human	Alto Paraíso, GO/Brazilian	2002	JF912190
Coast82	Human	Ivory Coast	1982	U54798
BeH422973	Human	Monte Alegre, PA	1984	JF912182
Bol 88/1999	Human	Santa-Cruz/Bolivia	2009	KF907504
BeH413820	Human	Porto Velho, RO	1983	JF912181
17DD	Vaccine	-	-	U17066
17D-P11B	Vaccine	-	-	JN811142

17D-P11C	Vaccine	-	-	JN811143
YFV case 1	Vaccine adverse event	Peru	-	GQ379162
17Db	Vaccine	-	-	X03700
YF-AVD2791-93F/04	Vaccine	-	-	DQ118157
17D-204	Vaccine	-	-	KF769015
17D RKI	Vaccine	-	-	JN628279
17D Flavimun WSL	Vaccine	-	-	JN628280
17D Flavimun TVX	Vaccine	-	-	JN628281
17D-P1	Vaccine	-	-	JN811140
17D-P11A	Vaccine	-	-	JN811141
Sanofi-Pasteur-17D-204	Vaccine	USA	-	JX503529
17Da	Vaccine	-	-	NC_002031
17DD-Brazil	Vaccine			DQ100292
17D-213	Vaccine	-	-	U17067
17D/Tiantan	Vaccine	-	-	FJ654700
YFV case 2	Vaccine adverse event	Peru	-	GQ379163
SAH177	Wesselsbron	-	-	EU707555
Wesselsbron	Wesselsbron	-	-	NC_012735
UgIL-30	Entebbe bat virus	-	-	DQ837641
Entebbe bat virus	Entebbe bat virus	-	-	NC_008718
MK7148	Sepik virus	-	-	DQ837642
Sepik virus	Sepik virus	-	-	NC_008719
TVP11767	*Alouatta seniculus*	Trinidad and Tobago	2009	HM582851
