# Supplementary material for: Phylogenomic analysis unravels evolution of yellow fever virus within hosts
Source: PLoS Negl Trop Dis. 2018 Sep 6;12(9):e0006738. doi: 10.1371/journal.pntd.0006738 (PMC6143276; doi:10.1371/journal.pntd.0006738)
Supplement: S3 Table — (PDF) [file pntd.0006738.s006.pdf]

Table S3. Phasing analysis for continuous streched iSNV

Streched iSNV GroupID	Sample	Pos	Ref > Alt	MuAF	CDS	Phased ?	Phased read ratio	Phased mutant read No.	Unphased mutant read No.	Wild-type read No.
1	S056	4278	A>G	0.10	NS2b	**No**	2.09%	49	2,295	26,896
1	S056	4356	A>G	0.07	NS2b					
1	S056	4547	T>G	0.06	NS2b					
2	S057	2392	A>T	0.08	E	**No**	0.00%	1	2,495	12,239
2	S057	2400	G>T	0.08	E					
3	S060	1096	C>T	0.26	E	**No**	9.10%	1,255	12,496	4,127
3	S060	1241	A>G	0.58	E					
4	S060	5412	A>G	0.15	NS3	**No**	6.60%	59	825	1,192
4	S060	5640	C>T	0.10	NS3					
5	S077	10360	G>A	0.15	3' UTR	**Yes**	96.47%	17,971	658	64,670
5	S077	10365	T>G	0.19	3' UTR					
5	S077	10367	C>T	0.19	3' UTR					
5	S077	10373	G>A	0.22	3' UTR					
5	S077	10398	T>C	0.39	3' UTR					
6	S078	10360	G>A	0.13	3' UTR	**Yes**	96.14%	19,615	787	65,163
6	S078	10365	T>G	0.16	3' UTR					
6	S078	10367	C>T	0.16	3' UTR					
6	S078	10373	G>A	0.19	3' UTR					
6	S078	10398	T>C	0.35	3' UTR					
6	S078	10425	A>G	0.50	3' UTR					
7	S079	10360	G>A	0.15	3' UTR	**Yes**	96.23%	16,133	632	58,637
7	S079	10365	T>G	0.18	3' UTR					
7	S079	10367	C>T	0.18	3' UTR					
7	S079	10373	G>A	0.22	3' UTR					
7	S079	10398	T>C	0.39	3' UTR					
8	S080	10360	G>A	0.16	3' UTR	**Yes**	95.38%	22,580	1,093	77,870
8	S080	10365	T>G	0.19	3' UTR					
8	S080	10367	C>T	0.19	3' UTR					
8	S080	10373	G>A	0.22	3' UTR					
8	S080	10398	T>C	0.41	3' UTR					
9	S112	624	C>T	0.09	M	**No**	0.70%	19	2,390	10,605
9	S112	669	T>C	0.09	M					
10	S112	1019	T>C	0.16	E	**No**	0.50%	28	5,547	5,418
10	S112	1243	A>G	0.12	E					
10	S112	1479	G>C	0.19	E					
10	S112	1504	A>C	0.21	E					
10	S112	1605	A>G	0.33	E					
11	S112	6632	C>G	0.54	NS4a	**No**	4.70%	155	3,118	1,099
11	S112	6820	T>A	0.24	2k protein					
12	S112	7627	A>G	0.06	NS4b	**No**	1.56%	413	26,062	4,373
12	S112	7649	C>T	0.36	NS5					
12	S112	7761	A>G	0.28	NS5					
13	S112	9452	G>T	0.23	NS5	**No**	3.50%	74	2,040	4,094
13	S112	9610	G>T	0.12	NS5					
14	S112	10360	G>A	0.10	3' UTR	**Yes**	93.34%	14,574	1,040	94,768
14	S112	10365	T>G	0.12	3' UTR					
14	S112	10367	C>T	0.12	3' UTR					
14	S112	10373	G>A	0.14	3' UTR					

14	S112	10398	T>C	0.27	3' UTR					
15	S126	7648	G>A	0.05	NS5	**No**	0.40%	1	204	1,358
15	S126	7876	G>A	0.07	NS5					
16	S126	10360	G>A	0.06	3' UTR	**Yes**	95.68%	12,815	579	91,430
16	S126	10365	T>G	0.08	3' UTR					
16	S126	10367	C>T	0.08	3' UTR					
16	S126	10373	G>A	0.09	3' UTR					
16	S126	10398	T>C	0.32	3' UTR					
16	S126	10425	A>G	0.51	3' UTR					
17	S157	8365	C>T	0.09	NS5	**No**	0.00%	29	31,610	124,479
17	S157	8394	T>C	0.09	NS5					
