# Supplementary material for: Phylogenomic analysis unravels evolution of yellow fever virus within hosts
Source: PLoS Negl Trop Dis. 2018 Sep 6;12(9):e0006738. doi: 10.1371/journal.pntd.0006738 (PMC6143276; doi:10.1371/journal.pntd.0006738)
Supplement: S4 Table — (PDF) [file pntd.0006738.s007.pdf]

Table S4 - Duration of viremia and evolutionary rates of YFV,ZIKV and DENV

Virus	Viremia duration	Descriptions in CDC (website)	Evolutiontion rate	Reference
YFV	3 days	approximately 3 days (https://www.cdc.gov/yellowfever/)	~4.2 × 10^{-4}	ref. 10
ZIKV	4-7 days	a few days to a week (https://www.cdc.gov/zika)	0.98 to 1.06 × 10^{-3}	ref. 6
DENV	3-7 days	highest in the first 3 or 4 days after onset of fever and then last for the next few days (https://www.cdc.gov/dengue/)	7.77 to 9.85 × 10^{-4}	ref. 25
DENV-1	-	-	7.773 × 10^{-4}	
DENV-2	-	-	8.600 × 10^{-4}	
DENV-3	-	-	9.851 × 10^{-4}	
DENV-4	-	-	9.712 × 10^{-4}	
