# Supplementary material for: Phylogenomic analysis unravels evolution of yellow fever virus within hosts
Source: PLoS Negl Trop Dis. 2018 Sep 6;12(9):e0006738. doi: 10.1371/journal.pntd.0006738 (PMC6143276; doi:10.1371/journal.pntd.0006738)
Supplement: S5 Table — (PDF) [file pntd.0006738.s008.pdf]

Table S5. Two primers set used for amplicon-sequencing

ID	L_primer	%GC*	TM$	Position#
Set1-1	TAAAACCCTGGGCGTCAAT	47.368	59.958	143
Set1-2	GGCACAAAGAAGGGAGTTCA	50	59.932	2140
Set1-3	CCTCATACATGGGCTTGACC	55	59.968	4108
Set1-4	GCTTCCATGCTCCTGGATAA	50	59.823	6054
Set1-5	ATTCGGTGGAACCGTCATT	47.368	59.814	8243
Set2-1	CGTTCGTTGAGCGATTAGC	52.632	59.213	83
Set2-2	CGTACTCTGCCCATTGCATA	50	59.345	967
Set2-3	ACGGTGGTCACGTCTCCT	61.111	58.645	1804
Set2-4	TCGTCCAAGACCCAAAGAAC	50	59.718	2719
Set2-5	GAGCGATGTTAGTGGGACAAG	52.381	59.389	3625
Set2-6	GGAGGAGTGGGGATGTTCTT	55	59.947	4567
Set2-7	GGCTCATAGAGCAAGAGCAAAT	45.455	59.682	5486
Set2-8	CGGAGCAAAAAGAGCATTG	47.368	59.184	6341
Set2-9	GGATTAAAGCCCAGCAATCA	45	59.687	7267
Set2-10	AACTGAGGGTGAGAGGACCTTA	50	58.905	8105
Set2-11	TGATGGGAAAGAGGGAAAAG	45	58.741	9004
Set2-12	CAGCAAAGCTTATGCAAACA	40	57.39	9914

ID	R_primer	%GC*	TM$	Position#
Set1-1	GCCAAACACGGTATGGATTC	50	59.839	2272
Set1-2	ATCAACTCTCCCTGCCACAC	55	59.652	4309
Set1-3	CAACCACACTGGCAAATCAC	50	59.619	6202
Set1-4	GCGTGCCTATCGGAAGAATA	50	59.847	8411
Set1-5	GTCTTTCCCTGGCGTCAATA	50	59.714	10731
Set2-1	TCAGGGGCCATTACAGTCA	52.632	59.663	1166
Set2-2	CGTTAGGTCATCTGCCACAA	50	59.338	2064
Set2-3	TGTTAGAGAAGGGGCACTCC	55	58.918	2899
Set2-4	GGCATCTCCTCCGTTGTTAAT	47.619	59.992	3741
Set2-5	CAGGTGCTCACATTCCTCAA	50	59.439	4656
Set2-6	CACCATTGGAGTGAGGGAAC	55	59.98	5644
Set2-7	ACCCTTCTTTGCCAGGAAAT	45	59.596	6586
Set2-8	ATTTCTGGGGCTTCCTCAA	47.368	59.248	7419
Set2-9	TGGAATTCCTGGAAAGAGGAT	42.857	59.542	8353
Set2-10	CCCAAGAGCCTCAAACTCAA	50	60	9118
Set2-11	CATCTAGCATGTCCTCAGTGGT	50	58.873	10090
Set2-12	GTGTTTTGTCATCCAAAGGTCT	40.909	57.732	10827

* %GC, GC content

$ TM, Primer melting temperature

position in reference genome
