# Supplementary material for: Phylogenomic analysis unravels evolution of yellow fever virus within hosts
Source: PLoS Negl Trop Dis. 2018 Sep 6;12(9):e0006738. doi: 10.1371/journal.pntd.0006738 (PMC6143276; doi:10.1371/journal.pntd.0006738)
Supplement: S6 Table — (PDF) [file pntd.0006738.s009.pdf]

Table S6: SNPs observed in YFV

				Reference					Our sequences			
ID of SNP	Samples	Sites in Genome	Gene	Sites in Gene	Nucleotides	Coden	Amino Acids	Sites in Coden	Sites in Amino acids	Major nucleotieds	Coden	Amino Acids
1	YF-BJ1/8D	900	M	417	T	GCT	A	3	139	C	GCC	A
2	YF-BJ1/8D	1974	E	999	C	CCC	P	3	333	T	CCT	P
3	YF-BJ1/8D	2352	E	1377	A	GCA	A	3	459	C	GCC	A
4	YF-BJ1/8D	3918	NS2a	408	C	ATC	I	3	136	A	ATA	I
5	YF-BJ1/8D	6463	NS4a	22	G	GTC	V	1	8	A	ATC	I
6	YF-BJ1/8D	7320	NS4b	432	A	GTA	V	3	144	G	GTG	V
7	YF-BJ1/8D	9783	NS5	2142	T	TCT	S	3	714	C	TCC	S
8	YF-BJ1/9D	900	M	417	T	GCT	A	3	139	C	GCC	A
9	YF-BJ1/9D	1974	E	999	C	CCC	P	3	333	T	CCT	P
10	YF-BJ1/9D	2352	E	1377	A	GCA	A	3	459	C	GCC	A
11	YF-BJ1/9D	3918	NS2a	408	C	ATC	I	3	136	A	ATA	I
12	YF-BJ1/9D	6463	NS4a	22	G	GTC	V	1	8	A	ATC	I
13	YF-BJ1/9D	7320	NS4b	432	A	GTA	V	3	144	G	GTG	V
14	YF-BJ1/9D	9783	NS5	2142	T	TCT	S	3	714	C	TCC	S
37	YF-BJ5/6D	900	M	417	T	GCT	A	3	139	C	GCC	A
38	YF-BJ5/6D	2352	E	1377	A	GCA	A	3	459	C	GCC	A
39	YF-BJ5/6D	3918	NS2a	408	C	ATC	I	3	136	A	ATA	I
40	YF-BJ5/6D	6463	NS4a	22	G	GTC	V	1	8	A	ATC	I
41	YF-BJ5/6D	9003	NS5	1362	C	AAC	N	3	454	T	AAT	N
42	YF-BJ5/10D	900	M	417	T	GCT	A	3	139	C	GCC	A
43	YF-BJ5/10D	2352	E	1377	A	GCA	A	3	459	C	GCC	A
44	YF-BJ5/10D	3918	NS2a	408	C	ATC	I	3	136	A	ATA	I
45	YF-BJ5/10D	6463	NS4a	22	G	GTC	V	1	8	A	ATC	I
46	YF-BJ5/10D	9003	NS5	1362	C	AAC	N	3	454	T	AAT	N
47	YF-BJ5/14D	900	M	417	T	GCT	A	3	139	C	GCC	A
48	YF-BJ5/14D	2352	E	1377	A	GCA	A	3	459	C	GCC	A
49	YF-BJ5/14D	3918	NS2a	408	C	ATC	I	3	136	A	ATA	I
50	YF-BJ5/14D	6463	NS4a	22	G	GTC	V	1	8	A	ATC	I
51	YF-BJ5/14D	9003	NS5	1362	C	AAC	N	3	454	T	AAT	N
52	YF-BJ2/16D	900	M	417	T	GCT	A	3	139	C	GCC	A
53	YF-BJ2/16D	2352	E	1377	A	GCA	A	3	459	C	GCC	A
54	YF-BJ2/16D	3470	NS1	1016	A	AAA	K	2	339	G	AGA	R
55	YF-BJ2/16D	3918	NS2a	408	C	ATC	I	3	136	A	ATA	I
56	YF-BJ2/16D	4278	NS2b	96	A	ATA	I	3	32	G	ATG	M
57	YF-BJ2/16D	6463	NS4a	22	G	GTC	V	1	8	A	ATC	I
58	YF-BJ2/16D	7320	NS4b	432	A	GTA	V	3	144	G	GTG	V
59	YF-BJ2/16D	8094	NS5	453	C	TCC	S	3	151	T	TCT	S
60	YF-BJ2/16D	8340	NS5	699	T	GTT	V	3	233	C	GTC	V
61	YF-BJ2/16D	10160	NS5	2519	T	GTT	V	2	840	C	GCT	A
62	YF-BJ4/13D	667	M	184	G	GAT	D	1	62	T	TAT	Y
63	YF-BJ4/13D	746	M	263	G	AGG	R	2	88	C	ACG	T
64	YF-BJ4/13D	840	M	357	G	CAG	Q	3	119	C	CAC	H
65	YF-BJ4/13D	900	M	417	T	GCT	A	3	139	C	GCC	A
66	YF-BJ4/13D	1881	E	906	C	GAC	D	3	302	T	GAT	D
67	YF-BJ4/13D	2352	E	1377	A	GCA	A	3	459	C	GCC	A

68	YF-BJ4/13D	3197	NS1	743	G	GGA	G	2	248	A	GAA	E
69	YF-BJ4/13D	3207	NS1	753	G	GTG	V	3	251	A	GTA	V
70	YF-BJ4/13D	3248	NS1	794	A	AAT	N	2	265	T	ATT	I
71	YF-BJ4/13D	3256	NS1	802	T	TGG	W	1	268	G	GGG	G
72	YF-BJ4/13D	3258	NS1	804	G	TGG	W	3	268	A	TGA	*
73	YF-BJ4/13D	3918	NS2a	408	C	ATC	I	3	136	A	ATA	I
74	YF-BJ4/13D	4206	NS2b	24	G	TTG	L	3	8	A	TTA	L
75	YF-BJ4/13D	4276	NS2b	94	A	ATA	I	1	32	G	GTA	V
76	YF-BJ4/13D	4598	NS3	26	C	CCT	P	2	9	T	CTT	L
77	YF-BJ4/13D	4954	NS3	382	G	GCA	A	1	128	T	TCA	S
78	YF-BJ4/13D	4955	NS3	383	C	GCA	A	2	128	T	GTA	V
79	YF-BJ4/13D	4956	NS3	384	A	GCA	A	3	128	C	GCC	A
80	YF-BJ4/13D	4977	NS3	405	T	AGT	S	3	135	G	AGG	R
81	YF-BJ4/13D	5015	NS3	443	A	GAA	E	2	148	G	GGA	G
82	YF-BJ4/13D	5018	NS3	446	T	GTG	V	2	149	C	GCG	A
83	YF-BJ4/13D	5066	NS3	494	T	GTG	V	2	165	C	GCG	A
84	YF-BJ4/13D	5225	NS3	653	G	AGA	R	2	218	C	ACA	T
85	YF-BJ4/13D	5277	NS3	705	G	GAG	E	3	235	A	GAA	E
86	YF-BJ4/13D	5302	NS3	730	G	GAT	D	1	244	T	TAT	Y
87	YF-BJ4/13D	5306	NS3	734	T	GTG	V	2	245	C	GCG	A
88	YF-BJ4/13D	5307	NS3	735	G	GTG	V	3	245	C	GTC	V
89	YF-BJ4/13D	6463	NS4a	22	G	GTC	V	1	8	A	ATC	I
90	YF-BJ4/13D	7213	NS4b	325	C	CCT	P	1	109	A	ACT	T
91	YF-BJ4/13D	7271	NS4b	383	T	ATT	I	2	128	A	AAT	N
92	YF-BJ4/13D	7320	NS4b	432	A	GTA	V	3	144	G	GTG	V
93	YF-BJ4/13D	7566	NS4b	678	T	ACT	T	3	226	C	ACC	T
94	YF-BJ4/13D	7627	NS4b	739	A	AAA	K	1	247	G	GAA	E
95	YF-BJ4/13D	8493	NS5	852	A	AGA	R	3	284	G	AGG	R
96	YF-BJ4/13D	8626	NS5	985	T	TTG	L	1	329	C	CTG	L
97	YF-BJ4/13D	10160	NS5	2519	T	GTT	V	2	840	C	GCT	A
98	YF-BJ4/13D	10356	NS5	2715	C	ATC	I	3	905	T	ATT	I
99	YF-FZ2/D16	900	M	417	T	GCT	A	3	139	C	GCC	A
100	YF-FZ2/D16	1540	E	565	G	GAC	D	1	189	C	CAC	H
101	YF-FZ2/D16	1634	E	659	G	GGG	G	2	220	T	GTG	V
102	YF-FZ2/D16	1846	E	871	A	ACT	T	1	291	C	CCT	P
103	YF-FZ2/D16	1865	E	890	A	TAC	Y	2	297	T	TTC	F
104	YF-FZ2/D16	1869	E	894	G	AAG	K	3	298	C	AAC	N
105	YF-FZ2/D16	1873	E	898	T	TGC	C	1	300	G	GGC	G
106	YF-FZ2/D16	2352	E	1377	A	GCA	A	3	459	C	GCC	A
107	YF-FZ2/D16	3751	NS2a	241	T	TCC	S	1	81	A	ACC	T
108	YF-FZ2/D16	3918	NS2a	408	C	ATC	I	3	136	A	ATA	I
109	YF-FZ2/D16	3948	NS2a	438	T	AAT	N	3	146	C	AAC	N
110	YF-FZ2/D16	6435	NS3	1863	T	GGT	G	3	621	C	GGC	G
111	YF-FZ2/D16	6463	NS4a	22	G	GTC	V	1	8	A	ATC	I
112	YF-FZ2/D16	6495	NS4a	54	G	AAG	K	3	18	A	AAA	K
113	YF-FZ2/D16	6637	NS4a	196	G	GGA	G	1	66	C	CGA	R
114	YF-FZ2/D16	7320	NS4b	432	A	GTA	V	3	144	G	GTG	V
115	YF-FZ2/D16	7634	NS4b	746	G	GGG	G	2	249	A	GAG	E
116	YF-FZ2/D16	9075	NS5	1434	G	ATG	M	3	478	A	ATA	I
117	YF-FZ2/D16	9077	NS5	1436	G	TGG	W	2	479	A	TAG	*
118	YF-FZ2/D16	9084	NS5	1443	G	GGG	G	3	481	A	GGA	G
119	YF-FZ2/D16	9966	NS5	2325	C	GAC	D	3	775	T	GAT	D
120	YF-FZ2/D16	10317	NS5	2676	G	AGG	R	3	892	A	AGA	R

121	YF-FZ4/D7	861	M	378	T	GTT	V	3	126	C	GTC	V
122	YF-FZ4/D7	900	M	417	T	GCT	A	3	139	C	GCC	A
123	YF-FZ4/D7	1662	E	687	C	CAC	H	3	229	T	CAT	H
124	YF-FZ4/D7	2038	E	1063	A	ATT	I	1	355	C	CTT	L
125	YF-FZ4/D7	2183	E	1208	C	ACA	T	2	403	A	AAA	K
126	YF-FZ4/D7	2352	E	1377	A	GCA	A	3	459	C	GCC	A
127	YF-FZ4/D7	2500	NS1	46	G	GGA	G	1	16	A	AGA	R
128	YF-FZ4/D7	2632	NS1	178	G	GTT	V	1	60	C	CTT	L
129	YF-FZ4/D7	2648	NS1	194	A	CAC	H	2	65	G	CGC	R
130	YF-FZ4/D7	2650	NS1	196	G	GAG	E	1	66	A	AAG	K
131	YF-FZ4/D7	2665	NS1	211	A	AGG	R	1	71	C	CGG	R
132	YF-FZ4/D7	2676	NS1	222	G	GAG	E	3	74	T	GAT	D
133	YF-FZ4/D7	2707	NS1	253	G	GAC	D	1	85	C	CAC	H
134	YF-FZ4/D7	3604	NS2a	94	G	GTA	V	1	32	T	TTA	L
135	YF-FZ4/D7	3649	NS2a	139	A	ACA	T	1	47	C	CCA	P
136	YF-FZ4/D7	3664	NS2a	154	G	GTG	V	1	52	T	TTG	L
137	YF-FZ4/D7	3665	NS2a	155	T	GTG	V	2	52	G	GGG	G
138	YF-FZ4/D7	3918	NS2a	408	C	ATC	I	3	136	A	ATA	I
139	YF-FZ4/D7	4490	NS2b	308	C	GCC	A	2	103	T	GTC	V
140	YF-FZ4/D7	4621	NS3	49	T	TGT	C	1	17	G	GGT	G
141	YF-FZ4/D7	4646	NS3	74	A	TAT	Y	2	25	T	TTT	F
142	YF-FZ4/D7	4838	NS3	266	G	GGG	G	2	89	A	GAG	E
143	YF-FZ4/D7	4903	NS3	331	G	GTT	V	1	111	A	ATT	I
144	YF-FZ4/D7	4983	NS3	411	C	ACC	T	3	137	A	ACA	T
145	YF-FZ4/D7	4998	NS3	426	T	ATT	I	3	142	A	ATA	I
146	YF-FZ4/D7	6463	NS4a	22	G	GTC	V	1	8	A	ATC	I
147	YF-FZ4/D7	7312	NS4b	424	C	CAT	H	1	142	T	TAT	Y
148	YF-FZ4/D7	7313	NS4b	425	A	CAT	H	2	142	C	CCT	P
149	YF-FZ4/D7	7320	NS4b	432	A	GTA	V	3	144	G	GTG	V
150	YF-FZ4/D7	8112	NS5	471	G	GAG	E	3	157	C	GAC	D
151	YF-FZ4/D7	8385	NS5	744	A	ACA	T	3	248	T	ACT	T
152	YF-FZ4/D7	8424	NS5	783	A	ATA	I	3	261	G	ATG	M
153	YF-FZ4/D7	9225	NS5	1584	T	GGT	G	3	528	G	GGG	G
154	YF-FZ4/D7	9622	NS5	1981	C	CGA	R	1	661	T	TGA	*
155	YF-FZ6/D20	549	M	66	G	ACG	T	3	22	T	ACT	T
156	YF-FZ6/D20	900	M	417	T	GCT	A	3	139	C	GCC	A
157	YF-FZ6/D20	1074	E	99	A	GTA	V	3	33	G	GTG	V
158	YF-FZ6/D20	2352	E	1377	A	GCA	A	3	459	C	GCC	A
159	YF-FZ6/D20	3918	NS2a	408	C	ATC	I	3	136	A	ATA	I
160	YF-FZ6/D20	4395	NS2b	213	T	TCT	S	3	71	C	TCC	S
161	YF-FZ6/D20	4811	NS3	239	C	GCT	A	2	80	G	GGT	G
162	YF-FZ6/D20	5442	NS3	870	G	GAG	E	3	290	A	GAA	E
163	YF-FZ6/D20	5562	NS3	990	C	TTC	F	3	330	T	TTT	F
164	YF-FZ6/D20	6109	NS3	1537	T	TAT	Y	1	513	C	CAT	H
165	YF-FZ6/D20	6129	NS3	1557	G	AAG	K	3	519	A	AAA	K
166	YF-FZ6/D20	6463	NS4a	22	G	GTC	V	1	8	A	ATC	I
167	YF-FZ6/D20	6967	NS4b	79	C	CCA	P	1	27	T	TCA	S
168	YF-FZ6/D20	7320	NS4b	432	A	GTA	V	3	144	G	GTG	V
169	YF-FZ6/D20	8288	NS5	647	C	ACT	T	2	216	T	ATT	I
170	YF-FZ6/D20	8359	NS5	718	C	CTG	L	1	240	A	ATG	M
171	YF-FZ6/D20	8370	NS5	729	G	AGG	R	3	243	A	AGA	R
172	YF-FZ6/D20	8436	NS5	795	T	AGT	S	3	265	C	AGC	S
173	YF-FZ6/D20	9184	NS5	1543	T	TAC	Y	1	515	C	CAC	H

174	YF-FZ6/D20	9253	NS5	1612	G	GGG	G	1	538	T	TGG	W
175	YF-FZ6/D20	10422	NS5	+66	G	-	-	-	-	A	-	-
176	YF-FZ6/D20	10424	NS5	+68	A	-	-	-	-	C	-	-
177	YF-FZ6/D20	10428	NS5	+72	T	-	-	-	-	A	-	-
178	YF-FZ6/D20	10429	NS5	+73	A	-	-	-	-	T	-	-
179	YF-FZ6/D20	10432	NS5	+76	T	-	-	-	-	C	-	-
180	YF-FZ6/D20	10434	NS5	+78	A	-	-	-	-	T	-	-
181	YF-FZ6/D20	10435	NS5	+79	G	-	-	-	-	A	-	-
182	YF-FZ6/D20	10436	NS5	+80	A	-	-	-	-	G	-	-
183	YF-FZ6/D20	10439	NS5	+83	C	-	-	-	-	A	-	-
184	YF-FZ6/D20	10440	NS5	+84	C	-	-	-	-	A	-	-
185	YF-FZ6/D20	10441	NS5	+85	G	-	-	-	-	C	-	-
186	YF-FZ6/D20	10442	NS5	+86	G	-	-	-	-	C	-	-
187	YF-FZ6/D20	10444	NS5	+88	A	-	-	-	-	G	-	-
188	YF-FZ6/D20	10445	NS5	+89	T	-	-	-	-	G	-	-
189	YF-FZ6/D20	10447	NS5	+91	A	-	-	-	-	T	-	-
190	YF-FZ7/D15	443	C	323	T	GTA	V	2	108	C	GCA	A
191	YF-FZ7/D15	900	M	417	T	GCT	A	3	139	C	GCC	A
192	YF-FZ7/D15	1043	E	68	C	GCC	A	2	23	T	GTC	V
193	YF-FZ7/D15	2258	E	1283	C	ACA	T	2	428	G	AGA	R
194	YF-FZ7/D15	2303	E	1328	A	CAG	Q	2	443	G	CGG	R
195	YF-FZ7/D15	2334	E	1359	G	ACG	T	3	453	C	ACC	T
196	YF-FZ7/D15	2352	E	1377	A	GCA	A	3	459	C	GCC	A
197	YF-FZ7/D15	2704	NS1	250	G	GTA	V	1	84	T	TTA	L
198	YF-FZ7/D15	2736	NS1	282	G	AAG	K	3	94	T	AAT	N
199	YF-FZ7/D15	3578	NS2a	68	G	AGA	R	2	23	T	ATA	I
200	YF-FZ7/D15	3918	NS2a	408	C	ATC	I	3	136	A	ATA	I
201	YF-FZ7/D15	4329	NS2b	147	T	GAT	D	3	49	C	GAC	D
202	YF-FZ7/D15	5001	NS3	429	G	GTG	V	3	143	A	GTA	V
203	YF-FZ7/D15	5003	NS3	431	A	AAC	N	2	144	T	ATC	I
204	YF-FZ7/D15	5025	NS3	453	G	GGG	G	3	151	C	GGC	G
205	YF-FZ7/D15	5258	NS3	686	C	ACC	T	2	229	A	AAC	N
206	YF-FZ7/D15	5459	NS3	887	C	CCT	P	2	296	A	CAT	H
207	YF-FZ7/D15	6463	NS4a	22	G	GTC	V	1	8	A	ATC	I
208	YF-FZ7/D15	6648	NS4a	207	T	ATT	I	3	69	A	ATA	I
209	YF-FZ7/D15	7320	NS4b	432	A	GTA	V	3	144	G	GTG	V
210	YF-FZ7/D15	7707	NS5	66	A	CAA	Q	3	22	C	CAC	H
211	YF-FZ7/D15	8151	NS5	510	G	AAG	K	3	170	A	AAA	K
212	YF-FZ7/D15	8303	NS5	662	A	TAC	Y	2	221	G	TGC	C
213	YF-FZ7/D15	9304	NS5	1663	A	ATG	M	1	555	T	TTG	L
214	YF-FZ7/D15	9630	NS5	1989	T	GCT	A	3	663	C	GCC	A
215	YF-FZ7/D15	9818	NS5	2177	G	AGG	R	2	726	A	AAG	K
216	YF-FZ7/D15	9839	NS5	2198	G	AGA	R	2	733	A	AAA	K
217	YF-FZ7/D15	9863	NS5	2222	G	AGA	R	2	741	A	AAA	K
